# Depressive symptoms among Thai male seafarers during the COVID-19 pandemic: a cross-sectional study

**DOI:** 10.1186/s12889-023-15305-7

**Published:** 2023-03-13

**Authors:** Woraluk Jonglertmontree, Orawan Kaewboonchoo, Ikuharu Morioka, Plernpit Boonyamalik

**Affiliations:** 1 Department of Public Health Nursing, Faculty of Public Health, Mahidol University, 420/1 Ratchawithi Road., Ratchathewi, 10400 Bangkok, Thailand; 2 Graduate School of Health and Nursing Science, Wakayama Medical University, Mikazura 580, 641-0011 Wakayama, Japan

**Keywords:** Depression, Factors, Seafarer, Thai, Maritime

## Abstract

**Background:**

Prevalence of depressive symptoms among seafarers is higher than the general population because of their unique work conditions. Factors that can be changed must be considered and promptly addressed in order to decrease the prevalence of depression. This study aims to clarify the prevalence of depressive symptoms and its related factors among Thai seafarers in an effort to contribute to policies and to prevent depression among Thai seafarers.

**Methods:**

This cross-sectional study was conducted among 381 male seafarers working onboard ocean-going vessels of five Thai shipping companies. The questionnaire items comprised of personal factors, working factors and depressive symptoms. Depressive symptoms were assessed using the Thai version of the Patient Health Questionnaire-9. First, the chi-square test was used for univariate analysis. Then, variables significantly associated by the chi-square test were used for multivariate logistic regression analysis (employing the stepwise method) as independent variables.

**Results:**

The average age of participants in this study was 36.4 years. Prevalence of depressive symptoms was 19.5%. One half of the participants (58.3%) reported subjective sleep problems, and most (75.1%) experienced poor coping behaviors. Two thirds (67.5%) were officers, and 10.1% of participants reported that they sometimes or never performed occupational safety behaviors. Regarding work environments, 62.2% reported that their work was disturbed from performing repetitive tasks. Multivariate logistic regression analysis showed two personal factors; sleep problems (Adjusted Odds Ratio (AOR) = 7.97, 95% Confidence interval (CI) = 3.52–18.05) and poor coping behaviors (AOR = 4.46, 95%CI = 1.61–12.34), and three working factors; job assignment (AOR = 2.50, 95%CI = 1.33–4.70), inadequate occupational safety behaviors (AOR = 4.51, 95%CI = 1.85–11.01) and performing repetitive task (AOR = 2.27, 95%CI = 1.16–4.45), were significantly associated with depression.

**Conclusion:**

During COVID-19 pandemic, 19.5% of Thai male seafarers had depressive symptoms. Personal and working factors including subjective sleep problems, poor coping behaviors, job assignment, performing inadequate occupational safety behaviors and performing repetitive tasks were risk factors of depressive symptoms among Thai male seafarers. Monitoring work environment rigorously and coping with work-related stress of the occupational safety behaviors program should be suggested.

## Introduction

Depression is the leading cause of illness and disability worldwide, causing individuals to experience poor function at work and in the family, which can be a risk factor for suicide [1]. According to occupational categories, although the prevalence of depressive symptoms among seafarers were varied from 12.3 to 49% in Chinese, Brazilian and English populations from 2017 to 2020 [2–4], this was a result of different types of depression measurements used, seafarer nationality, and differentiation in the times that data was collected. This means a higher prevalence of depressive symptoms than other occupations, e.g., farmers [5], garment workers [6] and nurses [7]. The unique work conditions of seafarers affecting their mental health can be categorized as individual and work environmental factors. The individual factors include age, experience, health status (high BMI, poor sleep and diabetics) and resilience [8]. The work environmental factors consist of two areas. Job demands comprise pressure from contractors/customers/time, working hours, ship department, job assignment, voyage episodes, period of seafaring, noise, and vibration. Job resources include instrumental support, team cohesion, shipboard caring, and effort-reward imbalance [8]. Such factors should be considered when investigating factors related to depression.

The global health crisis of the Corona Virus Disease 2019 (COVID-19) pandemic, declared in March of 2020 [9], has impacted seafarers’ working conditions. In response to measures to prevent the spread of infection, seafarers must be quarantined at ports for 14 days —during which they are isolated on ships, crew change is limited, and shipping service contracts are renewed monthly. Consequently, they experience extended periods of working and residing onboard ships, contributing negatively to their mental health [10]. Depression was found to be from 12.3 to 40.2% among seafarers [11, 12] during the COVID-19 pandemic. The prevalence did not change considerably, but has the possibility to increase if the COVID-19 pandemic continues. Factors that can change must be promptly addressed to decrease the prevalence of depression not only during the COVID-19 pandemic, but also in the future where we may experience similar challenges.

In Thailand, the first COVID-19 case was announced on January 13, 2020. A nationwide curfew was enforced until suspected and confirmed cases decreased, and was later lifted at the end of July 2020 [13]. From October to December 2020, the second wave of the outbreak began due to a new cluster related to shrimp markets in one province [14]. Immediate responses, instead of a national lockdown, were taken at three different levels depending on the outbreak and risk situation of the consecutive months beginning on January 2021. This included locking down certain provinces to facilitate active case detection in all suspected populations [15]. Therefore, seafarers were affected by either quarantine or isolation at ports in such provinces to prevent the spread of COVID-19. Their work at sea was extended even when they had no long term employment contract.

Besides, Thai seafarers have increased in number and constitute an important occupation due to the growth of maritime logistics in the Eastern Economic Corridor (EEC) established since 2018 officially. As to their mental health conditions, only one study reported that 77.19% experienced anxiety and stress caused by work conditions such as long periods of seafaring in 2019, before the COVID-19 pandemic [16]. Thai seafarers have had a very poor mental health situation in the past, and the COVID-19 pandemic may have exacerbated the situation. Clarifying the mental health of Thai seafarers and its related factors would be important during the COVID-19 pandemic, as a result of the COVID-19 era.

This study aimed to clarify the prevalence of depressive symptoms and its related factors among Thai seafarers during the COVID-19 pandemic. The results may help to address policies and practices for preventing depression among Thai seafarers.

## Materials and methods

### Study design and setting

This cross-sectional study was conducted among seafarers working onboard ocean-going vessels of five Thai shipping companies. All seafarers with different ranks and job categories were required to participate in the occupational safety training before going onboard in such companies. The participants in the training held from the 4th of January to the 31st of March 2021 were invited to participate in the current study.

At the time of this study, the restrictions of Thai seafarers due to the COVID-19 pandemic had not been eased. They were still required to quarantine at the seaport for two weeks.

### Participants, sample size, and eligible criteria

The targeted population was 38,066 registered Thai seafarers. The sample size was calculated based on a proportion formula with 95% confidence interval, 0.05 precision of estimation [17], and prior proportion rate of depressive symptoms at 20% [2]. The required sample size was 244. To obtain enough participants, the 146 sample was added (60% of required sample size) [18]. Finally, 390 samples were required.

The participants in this study were limited to male seafarers 18 years old or above, fluent in Thai and having onboard experience at least once. When the participants unable to complete the questionnaire on the training day, they were followed up for twice times through their supervisor.

### Data collection method and measurements

The self-administered questionnaire was assessed for validity using three expert panels and tested with 30 Thai seafarers at another shipping company. The questionnaire items are discussed below.

1. Personal factors.

Personal factors comprised age, underlying diseases, perceived health status when onboard oceangoing vessels, perceived susceptibility to COVID-19 when working on a ship, family history of mental health illness, stressful life event experiences, subjective sleep problems when working on a ship and coping behaviors.

Underlying diseases was verified as yes or no. If yes, the following diseases were also investigated: diabetes mellitus, hypertension, cardiovascular diseases, and others. Perceived health status was established as either good or poor. Perceived susceptibility to COVID-19 and family history of mental health illness were ascertained as either yes or no. Stressful life event experiences were determined as either yes or no. If yes, the following experiences were investigated: spouse/family member injured or died, pirate robbery/ assault, bad weather/storm risk for shipwreck, injury/ accident when working on a ship, friend injured/ had an accident/ died when working on a ship, and others. Subjective sleep problems were assessed using either yes or no.

Coping behaviors were evaluated using a short version assessment consisting of 28 items developed by Carver Brief Cope Inventory [19], translated to brief cope inventory in Thai with acceptable reliability and validity [20]. The question items included, how often the participants exhibited the behaviors when coping with problems. The answers were selected using a 4-point Likert scale in which 1 was, “I never perform this behavior”, 2 was, “I sometimes perform this behavior”, 3 was, “I usually perform this behavior” and 4 was, “I always perform this behavior”. Possible scores ranged from 28 to 112, and higher scores indicated good behavior. Scores were evaluated as low coping behaviors (scores: 28–56), moderate coping behaviors (57–84), and high coping behaviors (85–112) [21]. Cronbach’s α coefficient value was 0.85.

2) Working factors.

Working factors comprised of work conditions and environments.

Work conditions consisted of eight aspects: job assignment, job department, work pattern, period of seafaring, experience of seafaring, shipping services, social isolation, and occupational safety behaviors. Regarding job assignment, the job position (hierarchical seafarers’ work) was ascertained by selecting from the two items; ratings (non-officers) or officers. Job department (seafarers’ task on ship) was verified using three categories; engine, catering, or deck department. Concerning work pattern, work schedules of participants was determined using two patterns: shift work and daywork. Experience of seafaring was established by answering, “how many years have you worked as a seafarer?” Period of seafaring was ascertained by answering, “how many months have you worked on a ship currently?” Regarding shipping services, subjects were asked to choose from two services; tramp services (nonfixed shipping route and duration) or liner services (fixed shipping route and duration).

Social isolation was determined as a subjective experience of social isolation developed based on the social isolation concept of Zavaleta et al. [22] and divided as external and internal subjective experience. External subjective experience included three items concerning frequency of activities: contacting family members, coworkers/supervisors, and volunteers at a shipping company. Internal subjective isolation included four items regarding frequency of feeling: trust in co-workers, satisfaction with family members, coworkers/supervisors, and feelings of belonging with the shipping company. The responses included 1 = “not at all”, 2 = “sometimes” and 3 = “frequently”. The mean score was calculated using seven items, and a higher mean score indicated high social isolation. Cronbach’s α coefficient value was 0.70.

Occupational safety behaviors, referring to safety compliance, were measured using three items concerning frequency of compliance with rules and procedures, pre-use equipment check and appropriate use of personal protective equipment [23]. The responses included 1 = “never”, 2 = “sometimes” and 3 = “always”. The possible range was 3 to 9 points. Cronbach’s α coefficient value was 0.80.

Work environments were defined as seafarers’ perceptions of how much they were disturbed from work environments and evaluated using nine aspects. These included excessive noise and whole-body vibration from the ship as a physical work environments. Ergonomic work environments comprised lifting or pushing a heavy object, performing repetitive task, twisting the torso while working, standing or sitting continuously and limited workspace. Psychological work environments mainly refer to the amount of work to be done, pace of work, time pressure, and conflict at work inducing mental alertness, and arouse the experience of work-related strain [24]. In this study, high workload and low control in extreme time pressure, and conflict relationships with superior or coworkers on ship were measured. In all 9 items, responses were measured using a 3-point Likert scale: 1 = “disagree”, 2 = “neutral” and 3 = “agree.” Cronbach’s α coefficient value was 0.80.

3) Depressive symptoms.

Depressive symptoms were assessed using the Thai version of the Patient Health Questionnaire-9. (PHQ9). This is a 9-item self-reporting questionnaire designed to assess the current state of depressive symptoms with confirmed good reliability and validity [25] and has been widely used among several occupational groups in Thailand [26, 27] and other countries [28]. Each item has four responses concerning how often the participants experienced depressive symptoms within two weeks. The 0 to 3 scores referred to “never” to “always.” Possible scores ranged from 0 to 27 and were classified as no depression (0 to 8), mild depression (9 to 14), moderate depression (15 to 19), and severe depression (20 to 27). The standard score for experiencing depression equaled 9 points [25]. Cronbach’s α coefficient value was 0.90.

### Statistical analysis

Descriptive statistics were performed to determine prevalence regarding depressive symptoms, personal factors, and working factors. Those having a PHQ score of 9 or higher were considered to have depression. The answers to question items were classified as dichotomous variables to examine related factors to demonstrate the presence or absence of relevant factors rather than quantitatively. Dichotomous variables are discussed below. Low and moderate coping behaviors were categorized to the poor coping behavior group, and high coping behavior to the good coping behavior group. Job departments were coded in two variables. The first variable was divided as “catering/engine department” and “deck department”, and the second variable was divided as “catering/deck department” and “engine department”. Period of seafaring was divided to > 6 months and ≤ 6 months. Experience of seafaring was divided as > 10 years and ≤10 years. Social isolation, based on the average score (1.67), was divided as the “yes” group with scores ranging from 1.68 to 3.00 and the “no” group with scores ranging from 1 to 1.67. Occupational safety behaviors were divided as the “always” group (score 9) and “sometimes/never” group (score 8 or less). Responses of work environments were divided as yes (agree/neutral) and no (disagree).

The chi-square test was used for univariate analysis. Variables that were significantly associated at the chi-square test among personal and working factors were used for multivariate logistic regression analysis (using the stepwise method) as independent variables. Hosmer and Lemeshow goodness of test and Spearman’s rank test were used to check model fitness and multicollinearity, respectively. The results were revealed as adjusted odds ratios (AOR) and 95% confidence intervals (95%CI).

For statistical analyses, SPSS V.18 Software was used. P-value was set at < 0.05 to determine statistical significance.

### Ethics approval

The study was approved by the Ethics Committee for Human Research of the Faculty of Public Health, Mahidol University, Thailand (No. MUPH 2020 − 150 and protocol No. 120/2563). All procedures were performed in accordance with the Declaration of Helsinki. Written informed consent was obtained from all participants after they had been informed of the objectives, benefits, data collecting method and confidentiality agreement. Participants were right to withdraw from the study at any stage. No data in the questionnaires were traced back to any individual or shipping company to keep confidentiality.

## Results

### Characteristics of personal factor sand the prevalence in depressive symptoms

Of 390 invited participants in the training, 382 seafarers completed the questionnaires. One outlier participant was eliminated; thus, 381 were analyzed in this study, making a response rate of 98%.

As shown in the Table 1, the average age of participants in this study was 36.4 years. Altogether 7.1% had underlying diseases, among which hypertension was the most prevalent, 8.7% had poor perceived health status when working on a ship, 7.9% had perceived susceptibility to COVID-19 infection, 1.6% had family history of mental health illness, 37.3% had stressful life event experiences and 58.3% of had subjective sleep problems. Most (75.1%) had poor coping behaviors.

Concerning depressive symptoms, mild levels were the most prevalent (16.3%), whereas approximately 3% exhibited moderate to severe depressive symptoms. Prevalence regarding depressive symptoms among Thai seafarers was 19.5%.

**Table 1:** Characteristics of personal factors and prevalence of depressive symptoms

Characteristic of personal factors		N (%)
Age (years)	Mean (SD)	36.4 (9.6)
Underlying diseases	Yes	27 (7.1)
	Diabetes mellitus	7 (12.7)
	Hypertension	26 (47.3)
	Cardiovascular diseases	4 (7.3)
	Others	18 (32.7)
Perceived health status	Poor	33 (8.7)
Perceived susceptibility to COVID-19 infection	Yes	30 (7.9)
Family history of mental health illness	Yes	6 (1.6%)
Stressful life event experiences	Yes	142 (37.3%)
	Spouse/family member injured or died	106 (26.9%)
	Pirate robbery/assault	20 (5.1%)
	Bad weather/storms risk for shipwrecks	87 (22.1%)
	Injury/accident when working on ship	62 (15.7%)
	Friend injured/had an accident/died when working on ship	91 (23.1%0)
	Other	28 (7.1%)
Subjective sleep problem	Yes	222 (58.3%)
Coping behaviors	Low	0
	Moderate	286 (75.1%)
	High	75 (19.7%)
Prevalence in depressive symptoms	No depression	307 (80.6)
	Mild depression	62 (16.3)
	Moderate depression	11 (2.9)
	Severe depression	1 (0.3)

### Characteristics of working factors

As shown in Tables 2 and 87.9% of participants had shift work, 67.5% were officers, 59.3% were working in the deck department, and 53.3% were engaged in tramp shipping services. The average period of seafaring and experience of work was 8.0 months and 7 years, respectively. Only 4.8% perceived social isolation and 10.1% sometime or never performed occupational safety behaviors.

Regarding work environments, 85.6% of participants experienced that their work was disturbed from excessive noise, 89.8% from whole body vibration from ship motion, 75.1% from lifting or pushing a heavy object, 62.2% from performing repetitive task, 70.1% from twisting the torso while working, 69.6% from standing or sitting continuously. and 65.6% from limited workspace. Regarding psychosocial work environment, 92.9% had high job demand and low control in extreme time pressure and 61.9% had conflict relationships with supervisor or coworkers.

**Table 2:** Characteristic of working factors

Working factors		N (%)
**Working conditions**		
Work pattern	Shift work	335 (87.9%)
	Day work	46 (12.1%)
Job assignment	Ratings (nonofficers)	124 (32.5%)
	Officers	257 (67.5%)
Job department	Engine	123 (32.3%)
	Catering	32 (8.4%)
	Deck	226 (59.3%)
Shipping services	Tramp services	203 (53.3%)
	Liner services	178 (46.7%)
Experience of work (years)	Mean (SD)	7.0 (7.3)
Period of seafaring (months)	Mean (SD)	8.0 (2.6)
Social isolation	Yes	18 (4.8%)
Occupational safety behaviors	Sometimes/Never	38 (10.1%)
**Work environments**		
Excessive noise	Yes	326 (85.6%)
Whole body vibration from ship motion	Yes	342 (89.8%)
Lifting or pushing a heavy object	Yes	286 (75.1%)
Performing repetitive task	Yes	237 (62.2%)
Twisting the torso while working	Yes	267 (70.1%)
Standing or sitting continuously	Yes	265 (69.6%)
Limited workspace	Yes	250 (65.6%)
High job demand and low control in extreme time pressure	Yes	354 (92.9%)
Conflict relationship with supervisor or coworkers	Yes	236 (61.9%)

### Factors associated with depression

Table 3 displays the factors significantly associated with depression. In the univariate analysis, six personal factors and seven working factors were associated with depression.

Multivariate logistic regression analysis showed two personal factors including sleep problem (AOR = 7.97, 95%CI = 3.52–18.05) and poor coping behavior (AOR = 4.46, 95%CI = 1.61–12.34), and three working factors including ratings job assignment (AOR = 2.50, 95%CI = 1.33–4.70), sometime/never perform occupational safety behaviors (AOR = 4.51, 95%CI = 1.85–11.01), and performing repetitive task (AOR = 2.27, 95%CI = 1.16–4.45) were significantly associated with depression. All factors were positively associated and meant to be the risk factors of depressive symptoms.

**Table 3:** Personal factors significantly associated with depression

Factor	COR^1^ (95%CI^2^)	p-value	AOR^3^ (95%CI)	p-value
**Personal factors**				
Underlying diseases: Yes	3.74 (1.67–8.39)	0.001		
Perceived health status: Poor	2.61 (1.22–5.58)	0.011		
Perceived susceptibility to COVID-19 infection: Yes	3.59 (1.66–7.77)	0.001		
Stressful life event experiences: Yes	2.33 (1.39–3.90)	0.001		
Subjective sleep problem: Yes	6.76 (3.25–14.07)	< 0.001	7.97 (3.52–18.05)	< 0.001
Coping behavior: Poor	4.22 (1.64–10.90)	0.001	4.46 (1.61–12.34)	0.004
**Working factors**				
**Work condition**				
Job assignment: ratings	1.78 (1.06-3.00)	0.029	2.50 (1.33–4.70)	0.004
Work pattern: Shift work	2.13 (0.81–5.59)	0.012		
Social isolation: Yes	5.78 (2.20-15.22)	< 0.001		
Occupational safety behaviors: Sometimes/Never	4.02 (1.97–8.09)	< 0.001	4.51 (1.85–11.01)	0.001
**Work environment**				
Performing repetitive task Yes	2.85 (1.55–5.25)	0.001	2.27 (1.16–4.45)	0.003
Twisting the torso while working: Yes	2.30 (1.21–4.38)	0.010		
Conflict relationships with supervisor or coworkers: Yes	2.40 (1.33–4.31)	0.003		

^1^crude odd ratios ^2^confidence interval ^3^adjusted odd ratios

## Discussion

This study was conducted to clarify the prevalence of depressive symptoms and their associated factors among Thai seafarers during the COVID-19 pandemic. The prevalence of depressive symptoms was 19.5%.

The associated personal factors included subjective sleep problems and poor coping behaviors. The associated working factors comprised rating of job assignment, sometime or never performing occupational safety behaviors, and performing repetitive tasks.

Participants were all Thai male seafarers, with an average age of 36.4 years, working as officers on decks and in shifts. When their depressive symptoms were assessed at the beginning of the second wave in Thailand, approximately 20% reported such symptoms. This percentage was higher than that of one study (12.3%) [29] reporting depressive symptoms among almost all nonofficer seafarers, with multi-ethnicities, using English and in oil tanker ships. This was lower than the percentages of two studies targeting male and female seafarers who were almost all officers, using English, in various types of ships from July to September 2020 (41.5%) [30], and Chinese seafarers in various types of ships from June to July 2020 (41.7%) [11].

A study in a Japanese general population, published in the second wave of the COVID-19 pandemic, showed depressive symptoms in 18.35% of people using the same instrument (PHQ-9) [31]. This prevalence was the same as this present study, but the cutoff point was different. The Japanese study used the cutoff point “greater than 10” and our study used one “greater than 9” based on the scale of PHQ9 Thai version. When we used the same cutoff point, the rate was 13.4% and less than the Japanese report. Those who obtained the information related to COVID-19 for less than one hour daily exhibited lower risk to have depression than those who obtained it for three hours or more daily [32]. Because this present study was conducted at the time when positive cases gradually increased in Thailand, seafarers may have hardly been aware of the severity of the disease and the difficulty in working and receiving COVID-19 pandemic information. Whether or not the pandemic crisis occurred, many Thai seafarers present depressive symptoms that cannot be overlooked. Immediate implementation of policies and practices is desired to prevent depression among Thai seafarers.

Subjective sleep problem was one factor increasing the risk of depressive symptoms in the present study. This concurs with a systematic review and meta-analysis study showing that sleep problems were associated with depression among healthcare professionals, in the general population, COVID-19 patients [33] and Chinese seafarers [11] during the COVID-19 pandemic. Not only during the COVID-19 pandemic but before the pandemic, sleep problem also associated with depressive symptoms [2]. Ship motion may induce sleep disturbance related to depressive symptoms [34]. An imbalance of work-rest hour on ship is often caused by specific work conditions. The overtime work and unfix work time introduced by the increased shift work and the restricted crew change during the COVID-19 pandemic would be associated with the depressive symptoms.

Coping behavior is an individual cognitive and behavioral ability to manage generating emotions in specific stressful situations [35]. In this study, poor coping behavior was one factor to increase the risk of depressive symptoms. This finding was consistent with the report of Fukase et al. [31] showing that negative coping behaviors such as behavioral disengagement and self-blame were associated with depression.

A review article reported that officers had the most stressful job [36]; conversely, another study showed ratings (non-officers) were more at risk for high stress level than officers [38]. The present study showed that differences in seafarers’ job assignments were associated with the risk of depressive symptoms. In this study, the rating job was positively associated with depressive symptoms. Ratings (nonofficers) may not be responsible for demonstrating leadership in emergencies [12]. Their level of education was low and their knowledge may be associated with the less effectiveness in preventing mental health illness than officers who were highly educated [37].

Sometimes or never performing occupational safety behaviors involved the risk of depressive symptoms. This could be explained by a health belief model due to the lack of comparative studies concerning the relationship between occupational safety behaviors and depressive symptoms. When a person takes an action to prevent disease, it would be preceded by knowledge, perceived susceptibility, perceived severity, cues to action, and self-efficacy [38, 39]. Seafarers, who sometimes or never performed occupational safety behaviors, were not likely to be fully aware of the risks of work-related diseases and consequently may be unable to prevent acquiring diseases.

Seafarers perceiving disturbance by performing repetitive task were at risk of depressive symptoms. It would be difficult to compare this finding with others because no study has examined repetitive tasks related to depressive symptoms. Seafarers often repeat the same tasks at sea for extended periods of time. Those who would feel stressed about their work would also have mental health symptoms [40, 41]. Of course, the possibility cannot be denied that those who have depressive symptoms may easily perceive the disturbances relevant to work.

Sleep problems, personal coping behaviors, job assignment, and work conditions were already identified in a scoping review concerning factors related to depressive symptoms [8]. Occupational safety behaviors are mentioned as a highly important feature in remote workplaces [42]. In the case of seafarers, the response to occupational accidents caused by inadequate occupational safety behaviors may have been delayed. Furthermore, because this study suggested that occupational safety behaviors may be related to depressive symptoms, measures centered on occupational safety behaviors are also recommended to prevent depression.

### Policies and practices to prevent depression

Regarding the results of this study, policy and practices at work environment and individual levels could be advocated. In promoting a desirable working environment for mental health, ship supervisors should follow the procedures of the Maritime Labour Convention, 2006 [43], tasks evenly based on job assignment, and monitor the work environment rigorously including work-rest hours onboard. These are necessary to reduce unfair feelings and repetitive tasks in each job assignment, and to address subjective sleep problems. In individual level, the scope of occupational safety training should be extended to the contents of psychological health. For instance, methods on coping with work-related stress, breathing exercises, and muscle relaxation techniques may be trained within the occupational safety program before onboard seafaring [31, 44, 45].

### Limitations

The present study encountered several limitations. Firstly, the results were based on participants’ self-reported depressive symptoms and may contain recall and reporting biases. Secondly, depressive symptoms may have been underestimated because almost all participants perceived themselves as being healthy. Thirdly, a causal interpretation between factors and depressive symptoms may made with cautions because this cross-sectional study was conducted during COVID-19 pandemic. Fourthly, the participants were limited to Thai male seafarers. Thus, the results may not represent other nationality populations in the maritime field.

## Conclusion

The prevalence of depressive symptoms was 19.5% of Thai seafarers during the COVID-19 pandemic. Personal and working factors including subjective sleep problems, poor coping behaviors, job assignment, performing inadequate occupational safety behaviors, and performing repetitive tasks were risk factors of depressive symptoms among Thai seafarers. Ship supervisors are expected to rigorously monitor their work environment and extend the scope of occupational safety behaviors in programs concerning coping with work-related stress.

## Data Availability

The datasets used and/or analyzed during the current study are available from the corresponding author on reasonable request.

## Abbreviations

COVID-19: Corona Virus Disease 2019
PHQ-9: Patient Health Questionnaire-9
COR: Crude Odds Ratio
AOR: Adjusted Odds Ratio
EEC: Eastern Economic Corridor
